# Field Density Variation and Metabolite Profiling Reveal Transcriptome Mechanisms Driving Salt-Responsive Root Flavone Accumulation in *Scutellaria baicalensis*

**DOI:** 10.3390/ijms27208996

**Published:** 2026-10-11

**Authors:** Zirun Lin, Yaxin Zhang, Chenjie Zhao, Kun Zhao, Denglin Ma, Tao Zhang, Manhong Wang, Hua Yao

**Affiliations:** 1 Key Laboratory of Xinjiang Phytomedicine Resource and Utilization, Ministry of Education, College of Life Sciences, Shihezi University, Shihezi 832003, China; 18805092725@163.com (Z.L.); 13565367801@163.com (Y.Z.); 17615627537@163.com (C.Z.); zk2643164423@163.com (K.Z.); 13040541539@163.com (D.M.); 2 Pharmaceutical Research Institute, Urumqi 830010, China; ztbbq@163.com; 3 Department of Poultry Science, Mississippi State University, Starkville, MS 39762, USA; mw2911@msstate.edu.cn

**Keywords:** *Scutellaria baicalensis*, baicalin, baicalein, wogonoside, wogonin, 4′-deoxyflavones, salt stress, planting density, transcriptome, WGCNA, NAC transcription factors

## Abstract

Root flavones, especially baicalin, baicalein, wogonoside, and wogonin, contribute substantially to the medicinal quality of *Scutellaria baicalensis* Georgi. In the arid and semi-arid regions where this crop is grown, salinized soils constrain establishment but reshape specialized metabolism. We used field density trials, salt-stress experiments, ultra-performance liquid chromatography-tandem mass spectrometry (UPLC-MS/MS), and time-series RNA-seq, followed by weighted gene co-expression network analysis (WGCNA), to ask whether agronomic practices and salt-induced transcriptional changes alter root flavone content. Planting density showed a clear yield-quality trade-off: higher density increased belowground biomass per unit area but reduced individual root growth and flavone content, with 150 plants·m^−2^ providing a practical balance between population yield and root quality under the tested Xinjiang field conditions. In established seedlings, 0.5% NaCl caused no visible damage over 24 h and specifically raised baicalin and baicalein at 12 h, whereas wogonoside and wogonin (the wogonin-type flavones) remained nearly unchanged. Time-series RNA-seq identified thousands of differentially expressed genes between 2 and 24 h. WGCNA identified a turquoise module (one of the color-named co-expression modules) that was enriched for genes involved in carbohydrate metabolism and cytoskeletal/microtubule processes. Salt-responsive NAC, MYB and WRKY transcription factors co-expressed with core structural genes (*PAL*, *4CL*, *CHS*, *CHI*); one candidate, *SbNAC86*, was up-regulated mainly at 24 h after the metabolite peak, consistent with a potential role in the later phase of the response rather than in triggering the initial accumulation peak. These findings outline the temporal dynamics of salt-induced branch-selective flavone accumulation and suggest candidate regulators for functional validation, with implications for quality-focused cultivation on saline marginal lands.

## 1. Introduction

*Scutellaria baicalensis* Georgi (Huang-Qin) is a widely used traditional Chinese medicinal herb. Its dried roots are used to clear heat, dry dampness, purge fire, remove toxins, stop bleeding, and calm the fetus. Modern pharmacological studies have demonstrated anti-inflammatory, antibacterial, antiviral, antioxidant, hepatoprotective, neuroprotective, and anticancer activities, which are largely attributed to root flavones [1,2,3,4,5,6,7,8]. The major root flavones—baicalin, baicalein, wogonoside, and wogonin—are bioactive 4′-deoxyflavones that lack a 4′-hydroxyl group on the B-ring [3,4,5,9,10,11] (reviewed in [6,7,8,12]). These four compounds are characteristic quality markers of *S. baicalensis* roots and contribute substantially to medicinal quality. Therefore, changes in their accumulation are directly relevant to both pharmacological quality and the environmental regulation of specialized metabolism. For example, the Chinese Pharmacopoeia (2020 edition) specifies baicalin as a quality-control marker with a minimum content of 9.0% [13]. They are also considered the principal bioactive constituents underlying the anti-inflammatory, antiviral, antioxidant, hepatoprotective, and neuroprotective activities of Huang-Qin roots [1,2,3,4,5,6,7,8].

The root-specific 4′-deoxyflavone branch is embedded in the general phenylpropanoid/flavonoid pathway. The common pathway provides precursors through PAL, 4CL, CHS, and CHI: PAL catalyzes the deamination of phenylalanine to cinnamic acid, 4CL activates hydroxycinnamic acids to CoA esters, CHS condenses CoA esters with malonyl-CoA to form chalcones, and CHI isomerizes chalcones to flavanones [9,10,14,15,16,17,18]. The root-specific 4′-deoxyflavone branch is then defined by *SbCHS-2* (chalcone synthase), *SbFNSII-2* (flavone synthase II), *SbF6H* (flavone 6-hydroxylase), and *SbF8H* (flavone 8-hydroxylase), followed by downstream glycosyltransferases that convert baicalein and wogonin into baicalin and wogonoside, respectively [9,10,14,15,16,17,18]. These enzymes distinguish the root-specific 4′-deoxyflavone route from the common flavonoid pathway, making them relevant to the branch-selective flavone response hypothesized here.

Environmental factors regulate plant secondary metabolism. Salinity induces broad transcriptional and metabolic reprogramming and integrates stress signaling with changes in primary and secondary metabolism [19]. Chloride-dominated salinity imposes ionic toxicity, osmotic stress, and oxidative stress and triggers extensive transcriptome reprogramming. Common salt-responsive programs include SOS and NHX ion transporters, ROS-scavenging systems, ABA/jasmonate signaling, and transcription factor families such as MYB, NAC, WRKY, bHLH, and ERF. In medicinal plants, reallocation of primary carbon and activation of phenylpropanoid/flavonoid metabolism often follow these early signaling events. Despite this regional relevance, the specific knowledge gaps remain: it is unknown whether salt stress affects *S. baicalensis* root flavone accumulation; whether the response is concentration- and time-dependent; whether it is branch-selective for baicalein-branch flavones (baicalin and baicalein) over wogonin-branch flavones (wogonoside and wogonin); when such a response peaks; and which transcription factors are associated with it. Xinjiang is an important medicinal-plant cultivation region with high solar radiation; however, soil salinization is widespread and can constrain crop establishment and productivity. Saline soils cover approximately 13.36 million hectares in Xinjiang, and about one-third of arable land is affected by varying degrees of salinization [20]. Salinized soils may also alter bioactive compound profiles [21]. We therefore tested the hypothesis that moderate, short-term chloride salinity—rather than an established agronomic strategy—would selectively induce baicalein-branch flavones (baicalin and baicalein) rather than wogonin-branch flavones in established *S. baicalensis* seedlings, that this response would occur within a defined time window, and that it would be associated with candidate transcription factors. This hypothesis was based on previous reports that abiotic stresses can elicit changes in secondary metabolite accumulation in medicinal species, although responses depend on species, genotype, tissue, stress intensity, and duration and are not universally positive [22,23,24,25,26].

In addition, planting density and salinity can both influence root yield and flavone content, but their effects on *S. baicalensis* have mainly been examined separately, and their interaction has not been tested in a factorial design [27,28,29,30]. To address these questions, we used two complementary experiments rather than a factorial density × salinity design. First, a field-density trial tested how crowding affects root yield and flavone content at 75, 150, and 225 plants m^−2^. Second, in a controlled salt-stress experiment, 45-day-old established seedlings were treated with 0.5% NaCl; root flavones were quantified by UPLC-MS/MS at 0, 2, 6, 12, 24, and 48 h, and transcriptomes were profiled by RNA-seq at 2, 6, 12, and 24 h [31]. The 2 and 6 h samples were selected to capture early signaling, 12 h coincided with the metabolite peak, 24 h captured late transcriptional remodeling, and 48 h was included only for metabolite profiling to assess whether the response persisted. The field trial provided agronomic context for quality plasticity, whereas the salt experiment was designed to identify candidate regulatory genes and pathways associated with the temporal flavone response. Baicalin and wogonoside are major quality-related root flavones in *S. baicalensis* [32]; therefore, understanding how environmental factors shape their accumulation is relevant to basic research and breeding [33]. By characterizing the temporal salt-induced flavone response and nominating candidate transcription factors, this study provides a foundation for future functional validation and for quality-focused cultivation of *S. baicalensis* on saline marginal lands.

## 2. Results

### 2.1. Planting Density Influences the Relationship Between Yield and Flavonoid Quality

**Figure 1:**
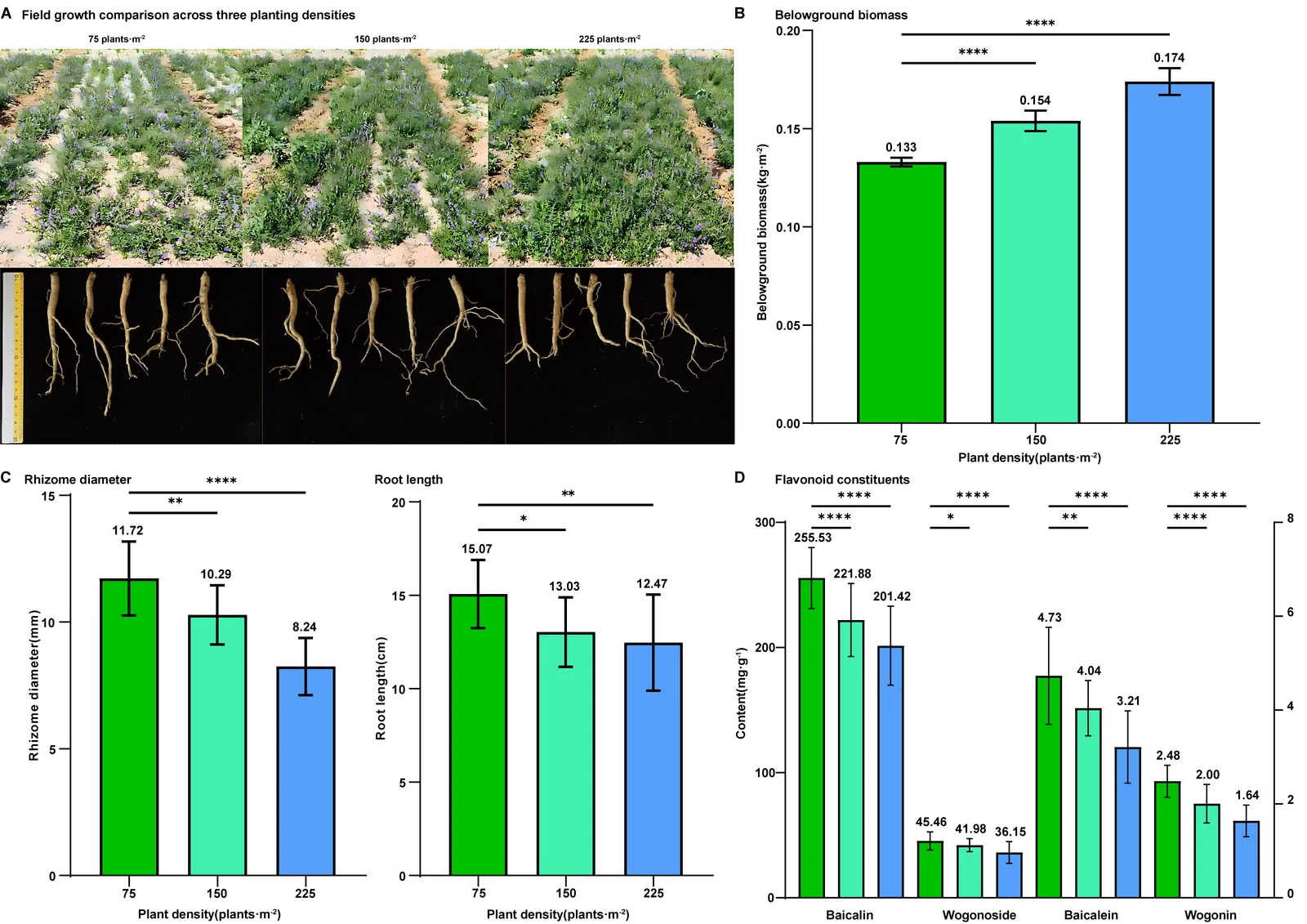
Agronomic plasticity under different planting densities. (**A**) Field growth comparison across three planting densities (75, 150, 225 plants·m^−2^). Among the three densities tested, 150 plants·m^−2^ provided a practical balance between belowground biomass production and root flavone content under the present field conditions. (**B**) Belowground biomass at different planting densities. (**C**) Rhizome diameter (left) and root length (right) under different planting densities. (**D**) Contents of four major root flavones in response to planting density. In (**D**), the left *y*-axis is for baicalin and wogonoside, and the right *y*-axis is for baicalein and wogonin. Values are mean ± SD. For (**B**–**D**), *n* = 3 plots per density. In (**C**), 10 plants per plot were measured for rhizome diameter and root length. In (**D**), roots from 10 plants per plot were pooled for flavone quantification. Asterisks above comparison bars indicate significant pairwise differences between planting densities based on one-way ANOVA followed by Tukey’s HSD test. Note: Significance levels: *, 0.01 < *p* ≤ 0.05; **, 0.001 < *p* ≤ 0.01; ****, *p* ≤ 0.0001.

At 75 plants·m^−2^, individual plants were largest and bore the most leaves, and the canopy did not close fully. At 225 plants·m^−2^, canopy closed early, and root biomass and baicalin concentration decreased. High density intensified intraspecific competition, causing stem elongation, leaf overlap, senescence of lower leaves, and reduced light transmittance. At 150 plants·m^−2^, the stand showed an intermediate canopy structure between the low- and high-density treatments (Figure 1A). Compared with 75 plants·m^−2^, belowground biomass per unit area increased, whereas individual root length, rhizome diameter, and root flavone concentrations were lower (Figure 1B–D). Root length and rhizome diameter were both diminished under the highest density, indicating that crowding constrained individual root development. Baicalin, wogonoside, baicalein, and wogonin were highest in roots from the lowest density and decreased progressively as density rose. High-density planting increased belowground biomass per unit area but reduced root flavone concentrations and individual root growth, revealing a trade-off between population-level biomass production and root quality.

Density reshapes the balance between population yield and root flavone accumulation. The medium density did not maximize individual-root flavone content but provided a practical compromise between biomass production and quality-related constituents. This field-scale plasticity sets the stage for the salt-stress experiments that follow, which dissect candidate regulatory mechanisms of flavone accumulation under a controlled abiotic stimulus. The density experiment should be interpreted as an agronomic context for quality plasticity, not as direct mechanistic evidence for the salt-response pathway. The soil agrochemical properties of the experimental field are summarized in Table S1 to document the field background and support reproducibility.

### 2.2. Moderate NaCl Stress Induces Baicalein-Branch Flavone Accumulation in Established Seedlings

**Figure 2:**
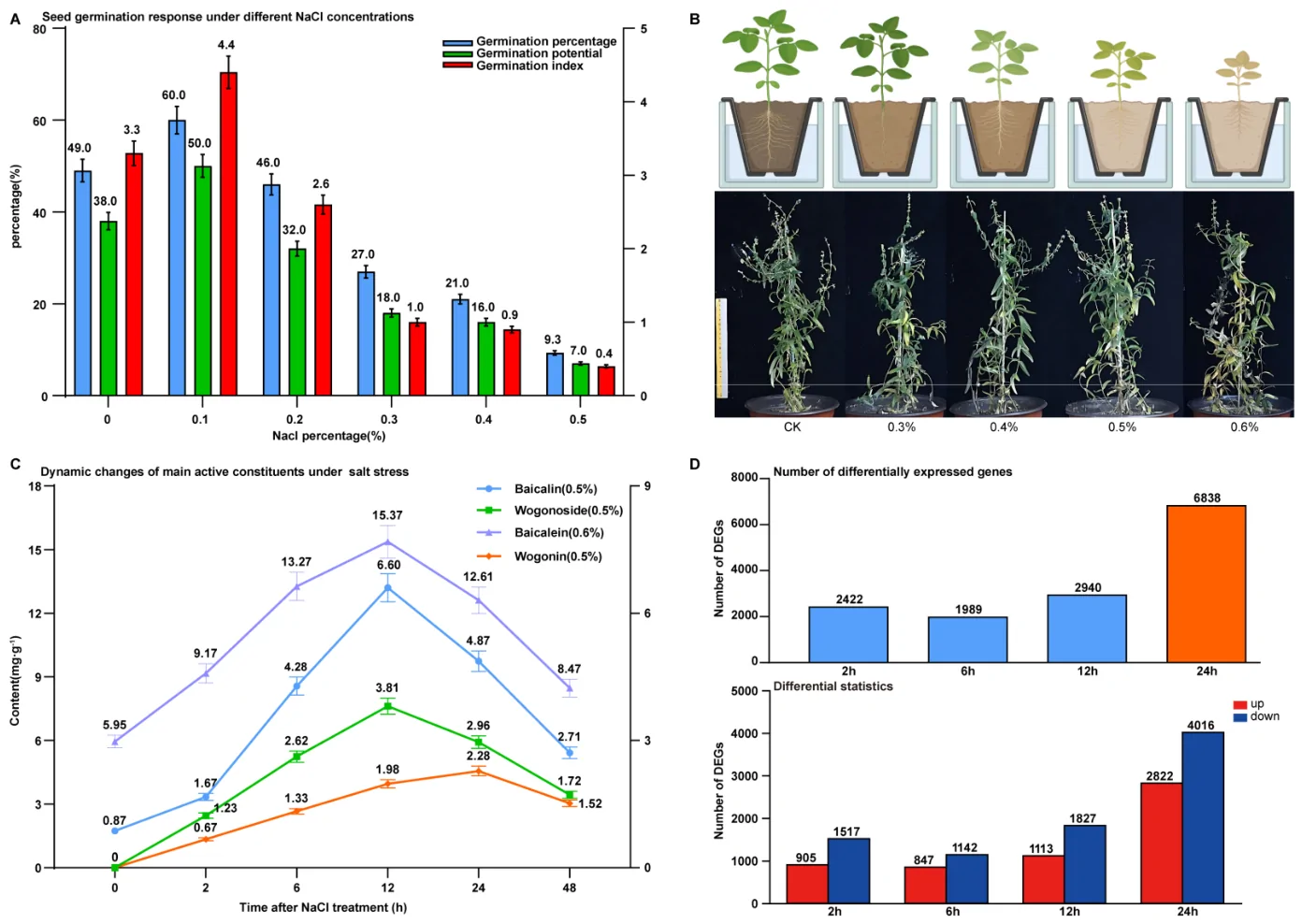
Moderate NaCl stress induces root flavone accumulation in *Scutellaria baicalensis*. (**A**) Seed germination percentage, germination potential, and germination index under 0–0.5% NaCl. *n* = 3 Petri dishes, 50 seeds per dish. Left *y*-axis, germination percentage and potential; right *y*-axis, germination index. (**B**) Representative morphology of seedlings after 15 d of salt treatment (CK and 0.3–0.6% NaCl). Each NaCl concentration and the control were maintained in physically separate pots; the schematic arrangement does not indicate a shared hydroponic vessel. *n* = 3 pots, six plants per pot. (**C**) Time-course of root baicalin, wogonoside, baicalein, and wogonin after a single 0.5% (*w*/*v*) NaCl treatment. Time-matched distilled-water controls are shown in Figure S7. Flavones were quantified at 0, 2, 6, 12, 24, and 48 h; RNA-seq was performed at 2, 6, 12, and 24 h. *n* = 3 pots, six plants per pot; each replicate was pooled from six plants. Left *y*-axis, baicalin and baicalein; right *y*-axis, wogonoside and wogonin. (**D**) Total number of differentially expressed genes (DEGs) at each time point (upper) and up- and down-regulated DEG counts (lower). Data are mean ± SD.

NaCl treatments were first screened for seed-germination tolerance. Germination parameters rose slightly at 0.1% NaCl, then dropped as concentration increased. At 0.1% NaCl, germination percentage and germination potential reached approximately 60.0% and 50.0%, respectively, compared with 49.0% and 38.0% in the control. Low salinity (0–0.2% NaCl) caused no marked inhibition, whereas concentrations of 0.3% NaCl and above progressively suppressed germination. At 0.5% NaCl, germination was strongly inhibited: germination percentage decreased to 9.3%, germination potential to 7.0%, and germination index to 0.4 (Figure 2A). Thus, 0.5% NaCl represents the upper limit of seed germination tolerance under our conditions, not an optimal germination concentration.

Seedling responses diverged from seed responses. After 15 d, plants under 0.5% NaCl appeared similar to controls, whereas 0.6% NaCl caused chlorosis, wilting, and stunting (Figure 2B). We therefore selected 0.5% NaCl as a moderate, short-term stress level for transcriptome analysis; 0.6% NaCl exceeded the tolerance range for sustained growth. Each treatment was applied to separate pots, and seeds were not pre-treated with NaCl.

Targeted flavone quantification showed that salt stress strongly affected root metabolite accumulation (Figure 2C). Control plants received 15 mL of distilled water once at the beginning of the treatment, corresponding to the volume applied to the NaCl-treated plants. Time-matched distilled-water control profiles are shown in Supplementary Figure S7. For clarity of presentation, Figure 2C shows the temporal profiles of the NaCl-treated plants, whereas the corresponding time-matched distilled-water controls are provided separately in Supplementary Figure S7. All comparisons with the salt-treated group were made against the corresponding time-matched controls. Baicalein-branch flavones displayed the strongest response. Baicalin and baicalein rose steadily and peaked synchronously at 12 h, reaching 6.60 and 3.81 mg·g^−1^, respectively. Relative to time-matched controls, baicalin was 9.21-fold higher and baicalein 4.39-fold higher. Wogonoside and wogonin showed weaker responses and different concentration/time optima. At 6 h, all four flavones dropped to 16–44% of control values, consistent with an early suppression before the 12 h increase. Because 0.5% NaCl produced a robust baicalin/baicalein induction while remaining tolerable for established seedlings over the short term, roots collected at 2, 6, 12, and 24 h under this condition were used for transcriptome sequencing. In addition, in Figure 2C, the 48-h time point was included only for metabolite profiling to determine whether the salt-induced flavone response persisted beyond 24 h; it was not included in the RNA-seq time series.

### 2.3. Time-Series RNA-Seq Reveals Progressive Transcriptomic Reprogramming Under 0.5% NaCl

Root transcriptomes of the salt-treated group (HQ; 0.5% NaCl) and time-matched controls (CK) were sequenced at 2, 6, 12, and 24 h. Differential expression analysis revealed 2422, 1989, 2940, and 6 838 DEGs at these time points (Figure 2D). The 24 h count rose to roughly 2.5 times the 12 h number, signaling a major transcriptional shift. Down-regulated DEGs outnumbered up-regulated DEGs at all four time points, suggesting broad suppression of basal cellular processes under prolonged salinity [34].

**Figure 3:**
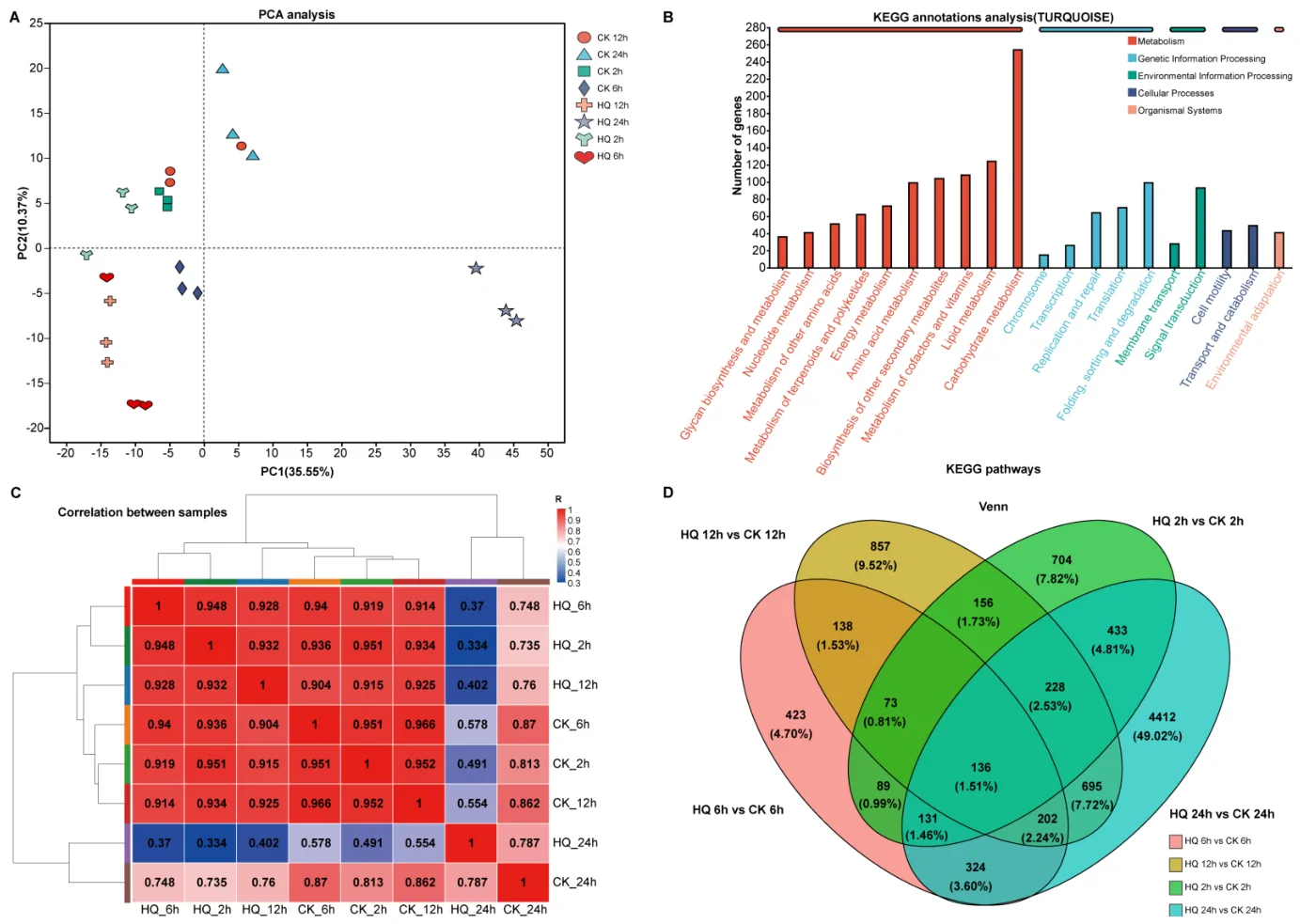
Transcriptomic responses and expression of flavonoid pathway genes in *S. baicalensis* roots under NaCl stress. (**A**) Principal component analysis (PCA) of transcriptomes from control (CK) and treated (HQ) samples at 2, 6, 12, and 24 h. (**B**) KEGG annotation analysis of genes in the turquoise WGCNA module. WGCNA modules are named by colors; the turquoise module is one of the co-expression modules identified by WGCNA. (**C**) Pearson correlation heatmap across samples. Grid values are correlation coefficients; red and blue indicate higher and lower correlations, respectively, and dendrograms show hierarchical clustering of sample replicates. (**D**) Venn diagram displaying the overlapping and unique DEGs among pairwise comparisons.

PCA separated salt-treated from control samples along PC1 (35.6% variance), and PC2 (10.37% variance) further revealed a time-dependent separation, with the 24 h treated group clearly diverging from earlier time points (Figure 3A). Moreover, sample distribution along the PC2 axis reflected a clear time-dependent trajectory, with the 24 h treated group conspicuously diverging from earlier time points. Sample-level Pearson correlation analysis further supported this temporal separation (Figure 3C). CK and HQ samples collected at 2, 6, and 12 h were highly correlated with one another (r = 0.904–0.966), whereas HQ_24 h showed markedly lower correlations with these early- and mid-stage samples (r = 0.334–0.578). CK_24 h remained relatively highly correlated with the earlier samples (r = 0.735–0.870) and with HQ_24 h (r = 0.787). Hierarchical clustering of the correlation matrix grouped the 2, 6, and 12 h samples together and separated HQ_24 h into a distinct branch, indicating that the 24-h salt treatment caused a pronounced transcriptomic reprogramming beyond the early response. Of all DEGs, 4412 were unique to the 24-h contrast, while only 136 appeared at all four time points (Figure 3D). Thus, the later time points contribute most of the transcriptomic change, not just an early response [34].

We used weighted gene co-expression network analysis (WGCNA) to identify modules of co-expressed genes (details in Section 2.4). In WGCNA, modules are named by colors such as turquoise, yellow, and blue. Each module eigengene represents the first principal component of the expression profiles of genes in that module. Here we focus on enrichment of the turquoise module rather than on total flavonoid correlations. KEGG enrichment analysis [35] of this module highlighted prominent roles for carbohydrate metabolism, followed by lipid metabolism, amino acid metabolism, biosynthesis of other secondary metabolites, membrane transport, signal transduction, and environmental adaptation (Figure 3B). These annotations support a stress-to-metabolism shift, in which salinity orchestrates nutrient reallocation, hormonal signaling such as zeatin signaling, and activation of specialized metabolic routes.

Transcriptomic data show that 0.5% NaCl activates distinct stress-responsive and specialized metabolic programs in roots. The coordinated upregulation of *PAL*, *4CL*, *CHS*, and *CHI* provides a plausible transcriptional basis for enhanced baicalin and baicalein accumulation; temporal repression of structural genes at 24 h suggests that activation is restricted to a tightly controlled window. This response is distinct from broader, late-stage adaptive reprogramming responses.

**Figure 4:**
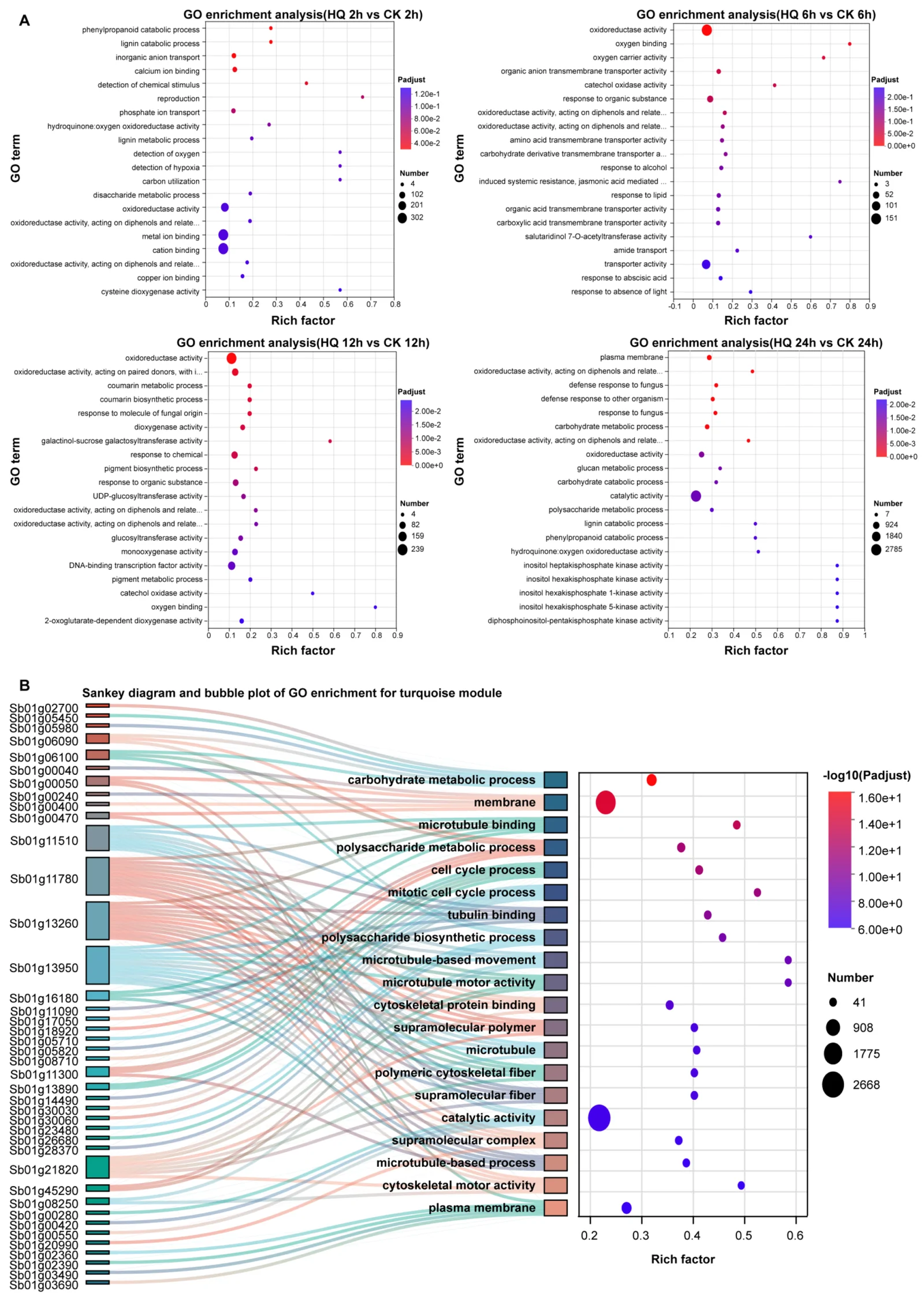
Gene Ontology (GO) enrichment analysis of DEGs and the turquoise module in *Scutellaria baicalensis.* (**A**) Bubble plots of GO enrichment for DEGs (HQ vs. CK) at 2, 6, 12, and 24 h. (**B**) Sankey diagram (left) linking genes to GO terms, and bubble plot (right) of GO enrichment for the turquoise module.

GO enrichment of DEGs from each time point (Figure 4A) showed that, across all sampling times, oxidoreductase activity (GO:0016491) was consistently enriched. This observation is biologically consistent with the biosynthesis of *S. baicalensis* flavonoids, which involves extensive redox reactions, particularly those catalyzed by cytochrome P450 monooxygenases and 2-oxoglutarate-dependent dioxygenases during the oxidative modification of the flavonoid backbone. Furthermore, early-stage GO terms were predominantly enriched in phenylpropanoid catabolic process and lignin catabolic process (at 2 h), while later stages (6 h and 24 h) showed a transition toward defense response to fungus, response to oxidative stress, and lipid transport. We also analyzed GO terms in the turquoise module. Genes within this module were significantly enriched in terms related to carbohydrate metabolic process, polysaccharide metabolic process, catalytic activity, as well as a highly distinctive suite of cellular structural and motility components, including microtubule binding, microtubule motor activity, microtubule-based movement, and cytoskeletal protein binding (Figure 4B). A possible link between this co-enrichment and metabolic channeling [36] is discussed in Section 3.2.

### 2.4. WGCNA Identifies a Turquoise Module Enriched in Flavonoid-Pathway Genes

**Figure 5:**
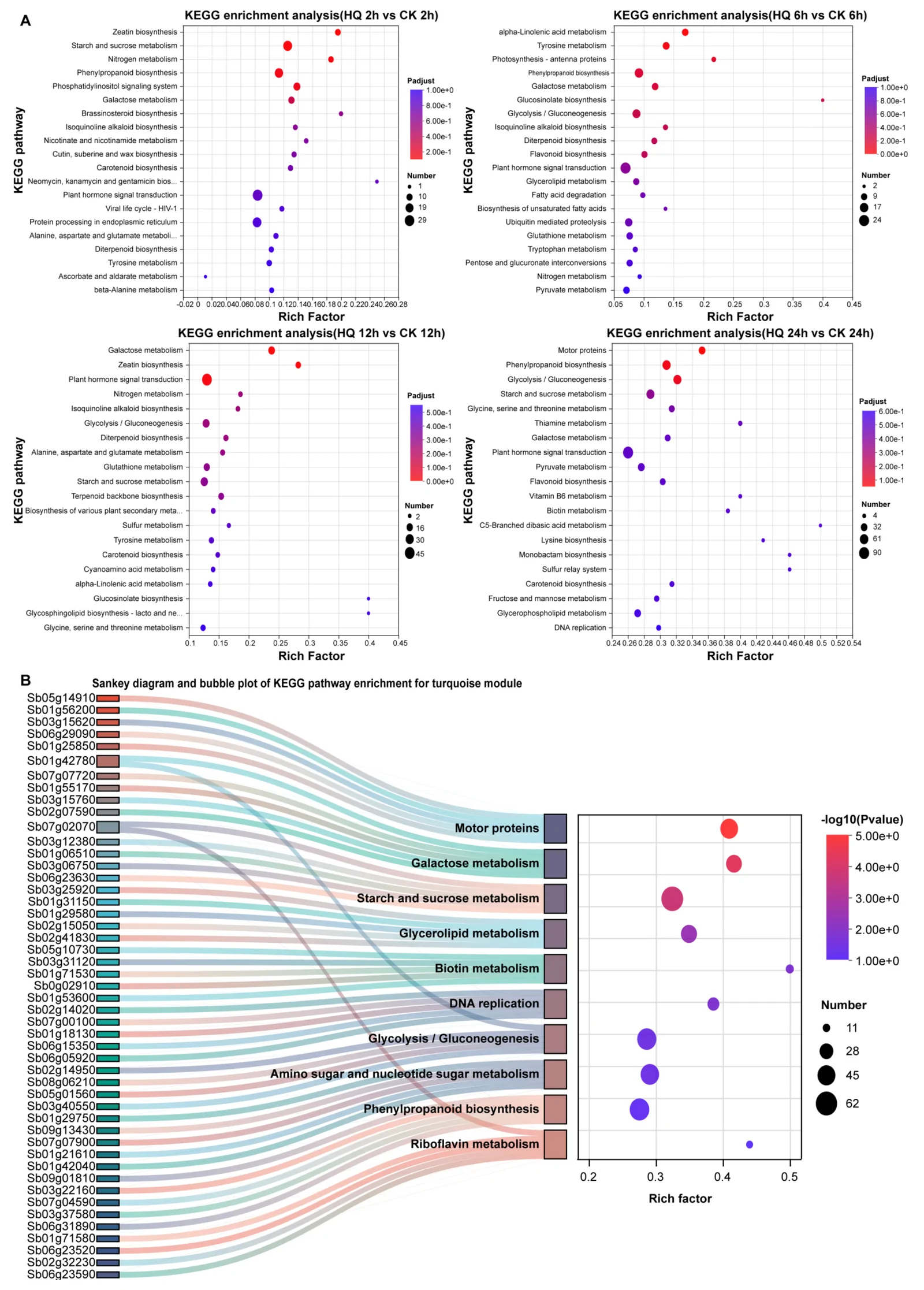
KEGG pathway enrichment analysis of differentially expressed genes (DEGs) and the turquoise co-expression module in *Scutellaria baicalensis.* (**A**) Bubble plots showing KEGG enrichment of DEGs between HQ and CK groups at 2, 6, 12, and 24 h. Rich factor, bubble size, and color represent the ratio of enriched DEGs to annotated genes, number of DEGs, and adjusted *p*-value, respectively. (**B**) Sankey diagram (left) linking hub genes from the turquoise module (WGCNA) to enriched KEGG pathways, and bubble plot (right) of the enrichment results. The turquoise module is one of the color-named WGCNA co-expression modules. Rich factor, bubble size, and color indicate the gene ratio, gene number, and −log10 (*p*-value), respectively.

**Figure 6:**
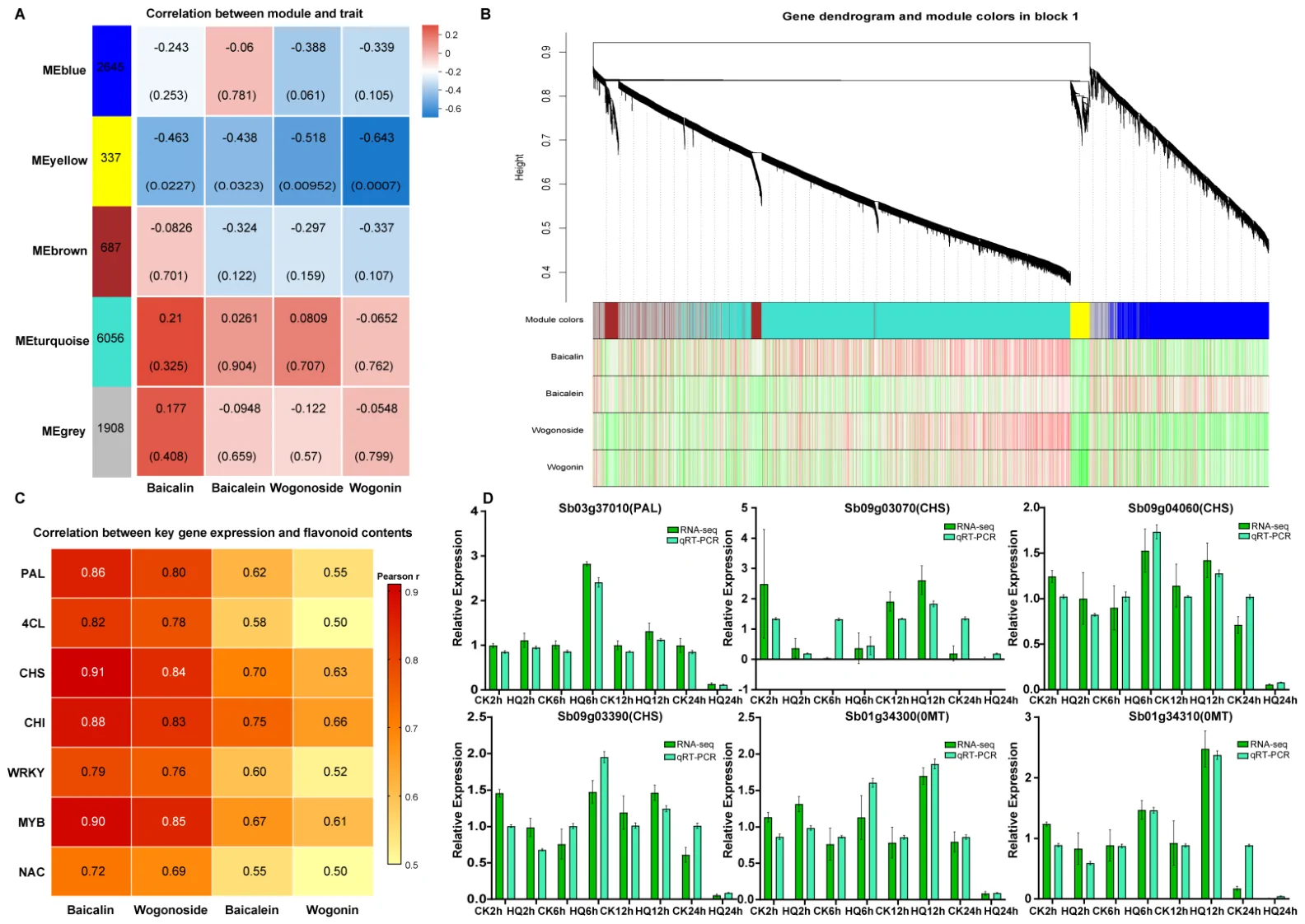
Weighted gene co-expression network analysis (WGCNA) and expression validation of key genes involved in the flavonoid biosynthetic pathway in *Scutellaria baicalensis*. (**A**) Heatmap of module-trait correlations. Rows represent co-expression modules identified by WGCNA, and modules are named by colors. Columns denote the four main flavonoids. Spearman correlation coefficients and corresponding *p*-values (in parentheses) are displayed in each cell. (**B**) Gene dendrogram and module color assignments. The color bands beneath the dendrogram represent distinct co-expression modules, and the bottom heatmap shows the accumulation profiles of the four flavonoids across samples. (**C**) Spearman correlation heatmap among the expression levels of key structural genes and transcription factors and the contents of the four flavonoids. The gradient from yellow to red indicates increasing positive correlation. (**D**) Relative expression levels of six candidate genes. Bar charts compare RNA-seq and qRT-PCR data in control (CK) and treatment (HQ) groups across different time points. Data are presented as mean ± SD.

WGCNA yielded a turquoise module (6056 genes; one of the color-named co-expression modules) enriched for carbohydrate and phenylpropanoid genes. KEGG analysis (Figure 5A) showed enrichment of DEGs in phenylpropanoid and flavonoid pathways, especially at 2 h and 6 h [9,37], as evidenced by high rich factors and small adjusted *p*-values. The turquoise module eigengene correlated weakly with baicalin and wogonoside, while a yellow module correlated negatively with wogonin (Figure 6A; |r| ≤ 0.43).

Genes in the turquoise module mapped to phenylpropanoid biosynthesis and to several primary metabolic pathways, including starch and sucrose metabolism, glycolysis/gluconeogenesis, and amino sugar and nucleotide sugar metabolism (Figure 5B). Hub genes such as *Sb05g14910* probably connect primary carbon metabolism with phenylpropanoid production [38]. Flavonoid synthesis draws phosphoenolpyruvate and erythrose-4-phosphate from glycolysis via the shikimate pathway [9,38]. Co-enrichment of glycolytic and sugar metabolism genes with phenylpropanoid genes argues for integrated carbon channeling. Flavonoid accumulation appears closely linked to, and energetically supported by, adjustments in primary carbohydrate metabolism.

KEGG enrichment of the turquoise module included phenylpropanoid and flavonoid biosynthesis, confirming that flavone-associated genes were organized into a metabolite-related co-expression module. Within the available annotation, ten candidate biosynthetic genes were identified in this module: three *PAL*, one *4CL*, four *CHS*, and two *CHI*. qRT-PCR validation of six selected genes supported the reliability of the RNA-seq expression trends.

### 2.5. Candidate Flavonoid Pathway Genes and Regulatory Correlations

WGCNA was used to find modules associated with the four major flavones [39,40,41,42]. A hierarchical clustering tree based on the topological overlap matrix defined modules by color (Figure 6B). As noted above, WGCNA modules are color-coded; MEyellow and METurquoise denote the module eigengenes of the yellow and turquoise modules, respectively. MEyellow correlated negatively with wogonin (Figure 6A), so this module may suppress wogonin accumulation; MEturquoise showed a weak positive correlation with baicalin and wogonoside. Spearman correlations were then calculated between structural genes (*PAL*, *4CL*, *CHS*, *CHI*), transcription factors (WRKY, MYB, NAC), and the four flavones (Figure 6C). Predicted transcription factor–target gene interactions are listed in Supplementary Table S3. *CHS* and *CHI* correlated most strongly with baicalin. TF regulation is examined in Section 2.6. qRT-PCR of six genes (Figure 6D) matched the RNA-seq data at every time point and treatment, confirming the transient up-regulation of *PAL* and *CHS* at 6 h.

### 2.6. Transcription Factors as Candidate Regulators of Flavonoid Biosynthesis

**Figure 7:**
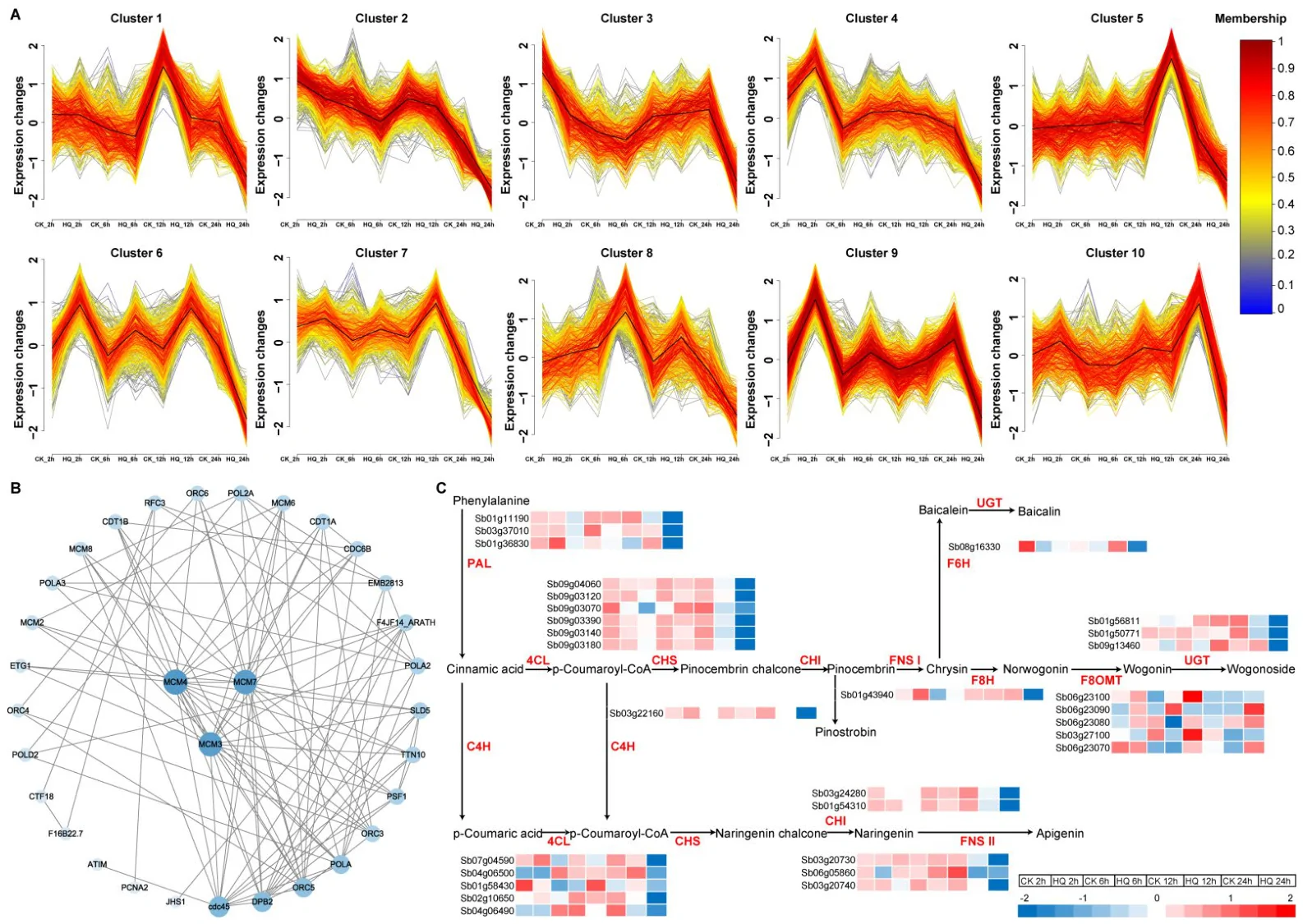
Temporal expression clustering analysis, interaction networks, and flavonoid biosynthetic pathways of related genes. (**A**) Mfuzz clustering of temporal expression profiles. *X*-axis, time points; *Y*-axis, expression levels. Color scale, membership values. Warmer colors denote higher importance. (**B**) Gene interaction network. Nodes, genes; edges, functional associations. (**C**) Flavonoid biosynthesis pathway. Enzymes are highlighted in red. Heatmaps display fold changes in corresponding genes/metabolites across indicated comparisons. Red denotes upregulation and blue downregulation.

Pathway mapping (Supplementary Figure S6) showed changes in central carbon and amino acid metabolism consistent with the KEGG results. Glycolysis, the TCA cycle, and amino acid metabolism were most affected, reflecting changes in energy production and carbon flow. Amino acid metabolism was enriched in the biosynthesis and degradation of branched-chain and aromatic amino acids. These processes are central to protein synthesis and nitrogen balance. Nucleotide metabolism also changed, together with alterations in lipid and cofactor/vitamin metabolism. This pattern indicates broad changes in carbohydrate, amino acid, lipid, and nucleotide metabolism. Mfuzz (version 2.66.0) grouped 5000 DEGs into ten temporal expression clusters (Figure 7A). Cluster 5 peaked early at 2 h; clusters 2 and 4 peaked at 12 h; clusters 1 and 3 declined over time, implying early signaling, mid-phase activation, and late remodeling [42]. Hierarchical clustering of phenylpropanoid and flavonoid structural genes (*PAL*, *4CL*, *CHS*, *CHI*, *FNS*, *OMT*, *UBGAT*) uncovered temporal order (Figure 7C). *Sb03g37010* (*PAL*) and *Sb09g03180* (*CHS*) were strongly up-regulated at 6 h and 12 h under HQ treatment, whereas most other structural genes were down-regulated by 24 h. This temporal pattern coincided with the 6–12 h metabolite accumulation window, indicating that activation of several flavone-biosynthetic genes preceded or accompanied the increase in root flavones.

We identified 2024 TF transcripts across 52 families. The most abundant were MYB, MYB-related, bHLH, ERF, NAC, WRKY, bZIP, C3H, GRAS, and HB-other, with MYB, NAC, and WRKY at the top—as expected for families with established roles in stress and secondary metabolism [38,43,44,45]. In a co-expression network comprising TFs, structural genes, and metabolites (Figure 7B), WRKY (*Sb03g41350*), MYB (*Sb04g02150*), and NAC (*SbNAC86*) showed strong connections to *PAL*, *4CL*, *CHS*, *CHI*, and to baicalin and baicalein [43,46]. These TFs are candidate regulators, but direct promoter binding and genetic validation are still required [38,47]. A working model is shown in Supplementary Figure S5. Mild salt stress then triggers a transcriptional response that peaks at 24 h (Figure 7A) and involves MYB, WRKY, and NAC TFs; these TFs were co-expressed with *PAL*, *4CL*, *CHS*, and *CHI* [43,46,48], and baicalin rises 9.21-fold while baicalein rises 4.39-fold. Moderate NaCl treatment (100 mM, approximately 0.58% *w*/*v*) has been shown to similarly enhance baicalin and baicalein production in hairy root cultures [49], consistent with the 0.5% NaCl condition used in this study.

### 2.7. Salt-Responsive Members of the SbNAC Family

Genome-wide transcription-factor family statistics for the *S. baicalensis* genome are summarized in the Supplementary Material. The MYB family was the largest (162 members), consistent with its well-established role in flavonoid regulation; the NAC family comprised 89 members. To identify salt-responsive candidate regulators of the flavone response, we profiled *SbNAC* expression across the salt-stress time course. Of the 48 *SbNAC* genes examined, 35 were differentially expressed under 0.5% NaCl, predominantly at 24 h. *SbNAC65*, *SbNAC30*, and *SbNAC22* showed the strongest late induction, whereas *SbNAC2* and *SbNAC51* were most strongly repressed (Supplementary Table S4). Together with salt-responsive MYB and WRKY members, these late-induced *SbNAC*s are nominated as candidate regulators of the sustained phase of flavone accumulation, to be tested functionally. Detailed phylogeny, conserved motifs, domain architecture, and chromosomal distribution are provided in Supplementary Figures S1–S3.

## 3. Discussion

### 3.1. Density and Salinity Operate Through Independent Pathways

Density changed root flavone content through resource competition, not stress signaling. Raising density from 75 to 225 plants·m^−2^ shortened and thinned individual roots and lowered all four flavones, even as it raised biomass per unit area. That inverse relationship is a classic yield-quality trade-off [50,51], similar to what has been reported in *Panax notoginseng* [51], though the exact magnitude varies by species and organ. Under the tested field conditions, a density of 150 plants·m^−2^ provided the most practical balance between biomass production and root flavone content among the three densities examined. Density changes light capture and source-sink balance, whereas salt influences ionic and osmotic stress, with opposing effects on flavonoid content; these contrasting patterns likely reflect different biological processes. Seed germination and established-seedling salt responses should be distinguished. The germination assay was used only as a tolerance screen; no metabolite or transcriptome analysis was performed on germinating seeds. All salt-induced flavone and transcriptome data were obtained from 45-day-old established seedlings treated with 0.5% NaCl. The 0.5% NaCl concentration almost completely inhibited seed germination but was tolerated by established seedlings and selectively induced baicalin and baicalein at 12 h. Therefore, the germination results define an upper tolerance boundary, whereas the established-seedling experiment addresses the salt-responsive flavone pathway.

In established seedlings, salt stress raised concentrations of baicalin and baicalein, which peaked at 12 h (Figure 2C). All four flavones showed a transient decrease to 16–44% of the corresponding control levels at 6 h before increasing at 12 h. This pattern may reflect a transient reorganization of metabolism during the early salt response, although the underlying mechanism was not directly examined in the present study [34]. The same NaCl concentration (0.5%) that almost completely blocked seed germination was tolerated by 45-day-old seedlings and increased baicalin and baicalein accumulation, indicating a strong developmental-stage dependence in salt sensitivity and metabolic response [52,53]. The response was branch-selective, with much stronger changes in baicalin and baicalein than in wogonoside and wogonin. This pattern suggests differential regulation of the two 4′-deoxyflavone branches; however, because pathway intermediates and metabolic flux were not measured, the specific regulatory step cannot be determined from the present data. Tests across salinity levels and growth stages are needed before any agronomic recommendations can be made in the future.

### 3.2. Transcriptional Dynamics Combine Primary Metabolism with Flavone Production

The RNA-seq time-series analysis conducted under 0.5% NaCl conditions revealed a transcriptional response pattern characterized by progressive and time-dependent features. DEG counts climbed from 2422 at 2 h to 6 838 at 24 h (Figure 2D). PCA separated treated from control samples along PC1, while PC2 resolved a time-dependent trajectory, with the 24 h group standing apart (Figure 3A). Early (2–6 h) and late (24 h) responses therefore appeared qualitatively different, with the latter encompassing broader shifts in metabolism, transport, and defense-related processes [54]. Similar time-dependent transcriptional remodeling under salinity has been reported in cotton [55], suggesting that progressive transcriptomic adjustment may occur in diverse plant species. WGCNA identified a turquoise module containing 6056 genes that was enriched in carbohydrate metabolism, phenylpropanoid-related processes, and flavonoid-associated pathways (Figure 5B), weakly correlated with baicalin and wogonoside (Figure 6A). Within this module, core structural genes were up-regulated at 2 h and 6 h and were sharply repressed by 24 h (Figure 7C). This early transcriptional pulse temporally preceded the 12-h increase in baicalin and baicalein, supporting an association between early activation of flavone-biosynthetic genes and subsequent metabolite accumulation. Subsequent repression at 24 h is consistent with a transient activation window, although its functional significance remains to be determined. qRT-PCR analysis of selected genes showed expression trends consistent with the RNA-seq data (Figure 6D).

The turquoise module showed significant enrichment of GO terms related to microtubules, together with genes involved in starch and sucrose metabolism and glycolysis (Figure 4B). This co-enrichment points to a possible metabolic channeling mechanism, in which cytoskeletal dynamics may spatially organize precursors and enzymes for efficient flavone production [36]. Flavonoid synthesis depends on metabolons—multienzyme complexes that cluster at the endoplasmic reticulum or in the cytosol—and their positioning depends on the cytoskeleton, particularly microtubules and actin filaments. Co-expression of cytoskeletal motor proteins with polysaccharide metabolic enzymes further suggests that the cytoskeleton may move precursors, cofactors, and biosynthetic enzymes to appropriate intracellular compartments, thereby channeling carbon from primary carbohydrate metabolism into flavonoid synthesis. The co-enrichment of cytoskeletal and carbohydrate-metabolism genes therefore raises the possibility that cytoskeletal organization contributes to metabolic channeling during flavone biosynthesis. However, the present transcriptomic data are correlative, and this hypothesis requires direct experimental validation. KEGG enrichment also highlighted hormone signaling, zeatin biosynthesis, and amino-sugar metabolism, suggesting coordinated adjustments in carbon and nitrogen metabolism [34]. Transcriptomic data are correlative, however, and direct measurements of sugars, organic acids, hormones, and enzyme activities would be needed to substantiate these interpretations.

A methodological limitation should be acknowledged. The structural genes we mapped belong to the general phenylpropanoid/flavonoid pathway. The root-specific 4′-deoxyflavone branch genes—*SbCHS-2*, *SbFNSII-2*, *SbF6H*, and *SbF8H* [10,17]—could not be unequivocally resolved because of paralog complexity and annotation limits. Although we observed salt-induced transcriptional activation of the broader pathway, the branch-specific metabolite shift (baicalein versus wogonin) cannot be directly assigned to differential expression of these particular branch genes from our RNA-seq data alone [17]. Isoform-specific qPCR or targeted resequencing would be needed to settle this point.

### 3.3. Late-Induced NACs Are More Likely to Act as Sustainers than Triggers: A Provisional Model

Among 52 transcription factor families, MYB, NAC, and WRKY dominated, matching their established roles in stress and secondary metabolism [38,43,45]. Of the 48 *SbNAC* genes profiled, 35 responded to 0.5% NaCl (FDR < 0.05), most being up-regulated at 24 h and a few repressed (*SbNAC2*, *SbNAC51*) (Supplementary Table S4). This late induction—well after the 12 h metabolite peak—implies that these *NAC*s participate in sustaining or modulating later phases rather than triggering the initial surge [56,57]. In the co-expression network (Figure 7B), certain WRKY (*Sb03g41350*), MYB (*Sb04g02150*), NAC (*Sb08g17730* designated *SbNAC86*) members showed connections with *PAL*, *4CL*, *CHS*, *CHI*, baicalin/baicalein contents. These correlations nominate them as candidates but do not demonstrate direct regulation. Whether these promoters are directly bound and functionally relevant remains to be tested [46]. Mfuzz clusters (Figure 7A) and metabolite profiles outline an early response at 2–6 h, a transient rise in core structural-gene expression, and peak baicalin/baicalein accumulation at 12 h. By 24 h, structural-gene expression declined while many TFs were up-regulated. These late-induced transcription factors may participate in attenuation of the early response and in subsequent metabolic adjustment; however, their specific regulatory functions require experimental validation. The late induction of NAC transcription factors may contribute to adjusting or sustaining the new metabolic state rather than initiating the flavone accumulation peak. *SbNAC65*, *SbNAC30*, and *SbNAC22* were strongly induced mainly at 24 h, after the 12-h metabolite peak. Future functional studies using overexpression or loss-of-function lines, combined with flavone measurements at later time points (36–72 h), could determine whether these *NAC* factors sustain or attenuate baicalein-branch flavone accumulation. The weak trait correlations further imply that the network is more complex than our current data can resolve.

## 4. Materials and Methods

### 4.1. Plant Materials and Field-Density Experiment

Seeds of *S. baicalensis* were obtained from the Experimental Field Station of Shihezi University (44°18′52″ N, 86°03′25″ E). Field cultivation was conducted in accordance with the local standard Technical Regulations for Cultivation of *Scutellaria baicalensis* Georgi (DB6528/T 193-2023) [58], issued by the Bayingolin Mongol Autonomous Prefecture Administration for Market Regulation, China. Seeds were sown in a nursery bed in April, and one-month-old seedlings were transplanted to the field in May at three planting densities: 75, 150, and 225 plants·m^−2^. Row spacing was fixed at 30 cm, whereas within-row plant spacing was adjusted according to the target planting density. This experiment employed a randomized complete block design with three replicates; each experimental plot measured 10 m in length and 3 m in width. Irrigation, weed management, and pest and disease control measures were uniformly implemented across all experimental plots. Roots were harvested in October of the same year, approximately five months after transplantation. Belowground biomass was determined after oven-drying the harvested roots at 65 °C to constant weight and was expressed on a ground-area basis. Root samples were oven-dried at 65 °C to constant weight, ground into powder, vacuum-sealed in amber bottles, and stored until flavone quantification. Root length and rhizome diameter were measured on freshly harvested roots using a vernier caliper. Three independent biological replicates per treatment were collected, immediately frozen in liquid nitrogen, and stored at −80 °C.

### 4.2. Salt Stress Treatment

NaCl, sodium hypochlorite, and ethanol were purchased from Sinopharm Chemical Reagent Co. (Shanghai, China). For the seed germination experiment, 50 seeds per treatment were surface-sterilized with 75% ethanol for 30 s and 2% sodium hypochlorite for 10 min, rinsed three times with distilled water, and placed on two layers of filter paper in 15-cm-diameter Petri dishes moistened with 15 mL of NaCl solution (0, 0.1, 0.2, 0.3, 0.4, or 0.5% *w*/*v*). Three dishes were used per treatment. At the beginning of the experiment, 15 mL of the corresponding NaCl solution or distilled water was added once to each Petri dish. Dishes were incubated at 25 °C in darkness for 7 d. No additional NaCl solution was added thereafter; evaporative water loss was compensated with distilled water based on dish weight. Germination was recorded daily when the radicle protruded through the seed coat, and Water loss was monitored by weighing, and the lost water was replenished with distilled water. Germination percentage, germination potential, and germination index were calculated according to the International Rules for Seed Testing (ISTA, 2020) [59]. Germination percentage (%) = (number of germinated seeds by day 7/total number of seeds tested) × 100. Germination potential (%) = (number of seeds germinated on day 4/total number of seeds tested) × 100 [60]. Germination index = Σ(Gt/Dt), where Gt is the number of germinated seeds on day t and Dt is the corresponding germination day [61]. For the pot experiment, seeds were not pre-treated with NaCl; they were surface-sterilized as described above and sown directly. Surface-sterilized seeds were sown in plastic pots (20 cm diameter × 30 cm height; approximately 9.42 L) filled with vermiculite and placed on black drainage trays (3 cm depth). A hole-sowing pattern was used with six holes per pot and 3–5 seeds per hole (approximately 18–30 seeds per pot). Plants were irrigated every 15 d with half-strength Murashige and Skoog (1/2 MS) nutrient solution (Beijing Solarbio Science & Technology Co., Ltd., Beijing, China; Cat. No. P1020), with supplemental distilled water supplied as needed before the salt-treatment period. Seven days after emergence, seedlings were thinned to one plant per hole (six plants per pot). Plants were not grown in a shared hydroponic vessel. Each treatment had three independent pots (biological replicates). Seedlings were 45 days old at treatment initiation. For the pot experiment, each NaCl concentration (0.3%, 0.4%, 0.5%, and 0.6%, *w*/*v*) was applied to separate pots. At the beginning of treatment, each pot received a single 15 mL application of the corresponding NaCl solution, delivered evenly around the base of the plants to ensure uniform exposure of the root zone; control pots received 15 mL of distilled water. No additional NaCl or water was added during the 48-h sampling period. The 0.5% (*w*/*v*) NaCl treatment was selected for metabolite profiling and RNA-seq because it induced baicalin and baicalein without causing visible damage. Roots were sampled at 0, 2, 6, 12, 24, and 48 h for flavone quantification; RNA-seq was performed at 2, 6, 12, and 24 h. Each replicate was pooled from six plants. The 48 h time point was included only for metabolite profiling to assess whether the salt-induced flavone changes persisted; it was not part of the RNA-seq time series. The schematic illustrations in the upper panel of Figure 2B were created using BioRender (BioRender, Toronto, ON, Canada; https://biorender.com; accessed on 3 October 2026) [62].

### 4.3. Quantification of Root Flavones

Baicalin, wogonoside, baicalein, and wogonin reference standards (purity ≥ 98%) were purchased from Chengdu Must Bio-Technology Co., Ltd. (Chengdu, China). Methanol, acetonitrile, and formic acid (HPLC grade) were purchased from Fisher Scientific (Waltham, MA, USA). Root flavones were quantified by ultra-performance liquid chromatography-tandem mass spectrometry (UPLC-MS/MS) using these authenticated standards. The UPLC-MS/MS system consisted of an ACQUITY UPLC system and an XEVO TQ-S triple-quadrupole tandem mass spectrometer (Waters, Milford, MA, USA; supplied by Shanghai Simaiou Analytical Instrument Co., Ltd., Shanghai, China). Chromatographic separation was performed on an ACQUITY UPLC BEH C18 column (50 mm × 2.1 mm, 1.7 μm; Waters, Milford, MA, USA) at 30 °C. Mobile phases were (A) 0.1% formic acid in water and (B) acetonitrile, with gradient elution at 0.3 mL·min^−1^. The gradient program was as follows: 0–1 min, 5% B; 1–5 min, 5–95% B; 5–7 min, 95% B; 7–7.5 min, 95–5% B; and 7.5–10 min, 5% B. The injection volume was 2 μL. Mass spectrometric detection was performed in negative electrospray ionization mode using multiple reaction monitoring (MRM). The MRM transitions were baicalin m/z 445.1 → 269.1, wogonoside m/z 459.1 → 283.1, baicalein m/z 269.0 → 169.0, and wogonin m/z 283.1 → 268.1 (Supplementary Table S5). Calibration curves were constructed separately for each analyte by plotting peak area against concentration, with coefficients of determination (R^2^) > 0.999 for all four compounds. Representative total-ion chromatograms of the four reference standards are shown in Supplementary Figure S4. For the salt-stress experiment, root flavones were quantified at 0, 2, 6, 12, 24, and 48 h after treatment. For the density experiment, roots were harvested in October, and their flavone contents were subsequently quantified. Dried root powder (0.5 g) was extracted with 10 mL of 70% methanol in an L6-180 ultrasonic cleaning machine (Shanghai Haozhuang Instrument Co., Ltd., Shanghai, China) for 30 min. The extract was centrifuged at 12,000× *g* for 10 min using a Centrifuge 5427R refrigerated centrifuge (Eppendorf AG, Hamburg, Germany) and then filtered through a 0.22 μm PVDF membrane filter (JINTENG, Tianjin Jinteng Experimental Equipment Co., Ltd., Tianjin, China) prior to UPLC-MS/MS injection. Recovery rates were 95.2–99.8%, and limits of detection were 0.05–0.2 μg/mL. Ultrapure water was prepared using an Exceed CC-40 laboratory ultrapure water system (Chengdu Aike Water Treatment Equipment Co., Ltd., Chengdu, China). An MS1602TS electronic analytical balance (0.01 mg readability; Mettler Toledo Instruments (Shanghai) Co., Ltd., Shanghai, China) was used for sample weighing. An electric thermostatic drying oven (model DHG-9070A; Shanghai Yiheng Scientific Instrument Co., Ltd., Shanghai, China) was used for sample drying.

### 4.4. RNA Extraction, Library Construction and Sequencing

Total RNA was extracted from 0.5% NaCl-treated and time-matched control roots at 2, 6, 12, and 24 h using the RNAprep Pure Plant Kit (Tiangen, Beijing, China) following the manufacturer’s protocol. RNA integrity, purity, and concentration were assessed using an Agilent 2100 Bioanalyzer (Agilent Technologies, Santa Clara, CA, USA), and only samples with RIN ≥ 8.0 were used for library construction. cDNA libraries were prepared using the NEBNext Ultra RNA Library Prep Kit (New England Biolabs, Ipswich, MA, USA). Three biological replicates were used per condition. Libraries were sequenced by BGI Genomics (Shenzhen, China) using an Illumina NovaSeq 6000 platform (Illumina, San Diego, CA, USA) with 150-bp paired-end reads. Each sample generated approximately 12 Gb of clean data with Q30 > 92% and mapping rates > 85% (Supplementary Table S6). Detailed quality-control and mapping statistics for all samples are provided in Supplementary Table S2.

### 4.5. Transcriptome Analysis

Raw reads were filtered using fastp [54] to remove adapters and low-quality reads. Clean reads were mapped to the *S. baicalensis* reference genome (ASM835377v2 [17]; Genome Warehouse: GWHAOTC00000000) using HISAT2 [63,64], and expression levels were quantified as TPM using RSEM [65]. Differential expression between salt-treated and time-matched control samples was analyzed with DESeq2 [66] using |log_2_ fold change| ≥ 1 and adjusted *p*-value (FDR) < 0.05. GO and KEGG enrichment analyses were performed with clusterProfiler [67]. Weighted gene co-expression network analysis (WGCNA) [39,40] was used to identify modules correlated with baicalin, wogonoside, baicalein, wogonin, and total flavonoid content. For WGCNA, a signed co-expression network was constructed with a soft-thresholding power of β = 4. Modules were defined by hierarchical clustering based on the topological overlap matrix, with a minimum module size of 30 genes and a merge cut height of 0.25 for combining similar modules. Before network construction, genes with a coefficient of variation (CV) below 0.1 were filtered out. Module eigengenes were calculated and correlated with the contents of baicalin, wogonoside, baicalein, and wogonin using Spearman’s rank correlation. Genes with module membership ≥ 0.3 were retained for subsequent module interpretation. Transcription factor families were classified using PlantTFDB [37] annotations. DESeq2 v1.30.1, WGCNA v1.70-3, and clusterProfiler v4.0 were used. The reference genome annotation (ASM835377v2) and PlantTFDB v4.0 were applied for transcription factor classification [37]. Temporal expression clustering was performed using the Mfuzz package (version 2.66.0; Bioconductor 3.20) [68] in R (version 4.4.1; R Foundation for Statistical Computing, Vienna, Austria). Raw reads were processed using fastp version 0.23.2 [54], mapped to the *S. baicalensis* reference genome using HISAT2 version 2.1.0 [63,64], and quantified using RSEM version 1.3.3 [65]. Differential expression analysis was conducted using DESeq2 version 1.30.1 [66], and GO and KEGG enrichment analyses were performed using clusterProfiler version 4.0 [67]. WGCNA version 1.70-3 [39,40] was used for co-expression network analysis.

### 4.6. Candidate Gene Annotation

Candidate pathway genes were assigned according to the reference genome annotation and, where possible, by homology to functionally characterized *S. baicalensis* flavone-biosynthetic enzymes. Because several enzyme families contain multiple paralogues with different branch specificities, the distinction between general flavonoid genes and root-specific 4′-deoxyflavone genes was treated cautiously. Definitive assignment of salt-responsive genes to *SbCHS-2*, *SbFNSII-2*, *SbF6H*, *SbF8H*, *SbUBGAT*, or *SbPFOMT* orthologues should be further confirmed using sequence identity, coverage, phylogeny, and functional evidence.

### 4.7. qRT-PCR Validation

To validate the RNA-seq data, six differentially expressed genes (one *PAL*, three *CHS*, and two *OMT* genes) were selected from the flavonoid-related pathway. Primers were designed using Primer Premier 5.0 (PREMIER Biosoft International, Palo Alto, CA, USA) [69] and are listed in Supplementary Table S7. Total RNA (1 μg) was reverse-transcribed into cDNA using the PrimeScript RT Reagent Kit (Takara, Osaka City, Japan). qRT-PCR was performed on a Roche LightCycler 480 system using SYBR Green I Master Mix (Roche, Basel, Switzerland). The *S. baicalensis* 18S rRNA gene was used as the internal reference according to previous studies. Relative expression was calculated using the 2^−ΔΔCT^ method [70]. Three biological replicates and three technical replicates were used for each sample.

### 4.8. Statistical Analysis

Quantitative data are presented as mean ± standard deviation (SD). Statistical significance among multiple groups was assessed by one-way ANOVA followed by Tukey’s HSD test [71] using GraphPad Prism (version 10.1.2 for Windows; GraphPad Software, San Diego, CA, USA, www.graphpad.com). Asterisks indicate significant differences at *p* < 0.05. For RNA-seq data, differential expression thresholds were |log_2_ fold change| ≥ 1 and adjusted *p*-value (FDR) < 0.05.

## 5. Conclusions

Planting density and short-term NaCl treatment affected root flavone accumulation in different experimental contexts. Increasing planting density increased belowground biomass per unit area but reduced individual-root growth and flavone content, with 150 plants·m^−2^ providing a practical balance under the tested field conditions. In established seedlings, 0.5% NaCl selectively increased baicalin and baicalein, with the strongest response observed at 12 h. Time-series transcriptome analysis identified an early response of flavone-biosynthetic genes and pronounced transcriptional reprogramming at 24 h. WGCNA further identified a turquoise co-expression module enriched in carbohydrate metabolism, cytoskeletal processes, and flavonoid-related genes. Several NAC transcription factors were induced predominantly after the metabolite peak, making them candidates for future functional validation. Together, these results describe the temporal and branch-selective nature of the salt response and provide candidate genes and pathways for further investigation of quality-related flavone regulation in *S. baicalensis*.

## Data Availability

The original contributions presented in this study are included in the article/Supplementary Material. Further inquiries can be directed to the corresponding author.

## Supplementary Materials

The following supporting information can be downloaded at: https://www.mdpi.com/article/10.3390/ijms27208996/s1.

## Abbreviations

The following abbreviations are used in this manuscript:

**cDNA**	Complementary DNA
**CHI**	Chalcone isomerase
**CHS**	Chalcone synthase
**GO**	Gene ontology
**4CL**	4-coumaroyl-CoA ligase
**KEGG**	Kyoto encyclopedia of genes and genomes
**mRNA**	Messenger RNA
**PAL**	Phenylalanine ammonia lyase
**PCR**	Polymerase chain reaction
**qRT-PCR**	Real-time quantitative PCR
**RNA**	Ribonucleic acid
**WGCNA**	Weighted Gene Co-expression Network Analysis
**ANOVA**	Analysis of variance
**BLAST**	Basic Local Alignment Search Tool
**CK**	Control (non-stressed samples)
**DEG**	Differentially expressed gene
**FDR**	False discovery rate
**HQ**	0.5% NaCl-treated group
**MRM**	Multiple reaction monitoring
**NCBI**	National Center for Biotechnology Information
**PCA**	Principal component analysis
**ROS**	Reactive oxygen species
**SD**	Standard deviation
**SRA**	Sequence Read Archive
**TF**	Transcription factor
**UPLC-MS/MS**	Ultra-performance liquid chromatography–tandem mass spectrometry
