# Supplementary material for: Field Density Variation and Metabolite Profiling Reveal Transcriptome Mechanisms Driving Salt-Responsive Root Flavone Accumulation in Scutellaria baicalensis
Source: Int J Mol Sci. 2026 Oct 11;27(20):8996. doi: 10.3390/ijms27208996 (PMC13704419; doi:10.3390/ijms27208996)
Supplement: Supplementary file 1 [file ijms-27-08996-s001.zip › Supplementary Figures S1-S7.pdf]

Supplementary Materials

Field Density Variation and Metabolite Profiling Reveal Transcriptome Mechanisms Driving Salt Responsive Root Flavone Accumulation in *Scutellaria baicalensis*

Zirun Lin, Yaxin Zhang, Chenjie Zhao, Kun Zhao, Denglin Ma, Tao Zhang, Manhong Wang, Hua Yao

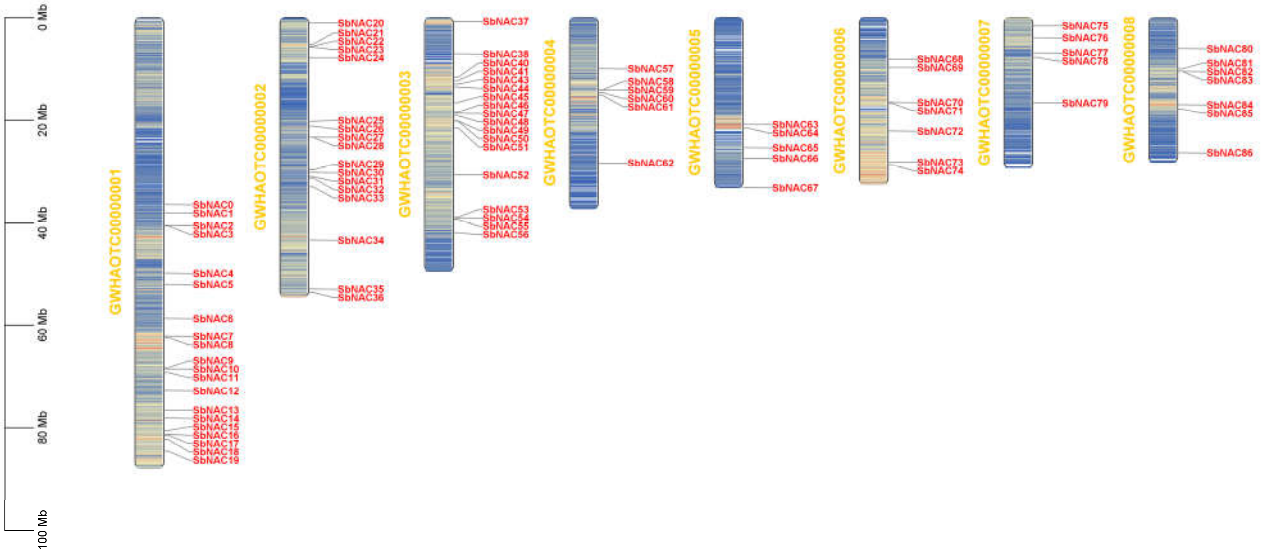

Supplementary Figure S1. Chromosomal distribution and physical mapping of the *SbNAC* gene family in *Scutellaria baicalensis*. 86 *SbNAC* genes were mapped onto the eight chromosomes. The colored bands on the chromosomes represent gene density, whereas the red labels and leader lines indicate the physical loci of the respective *SbNAC* genes. The scale bar on the left is in megabases (Mb).

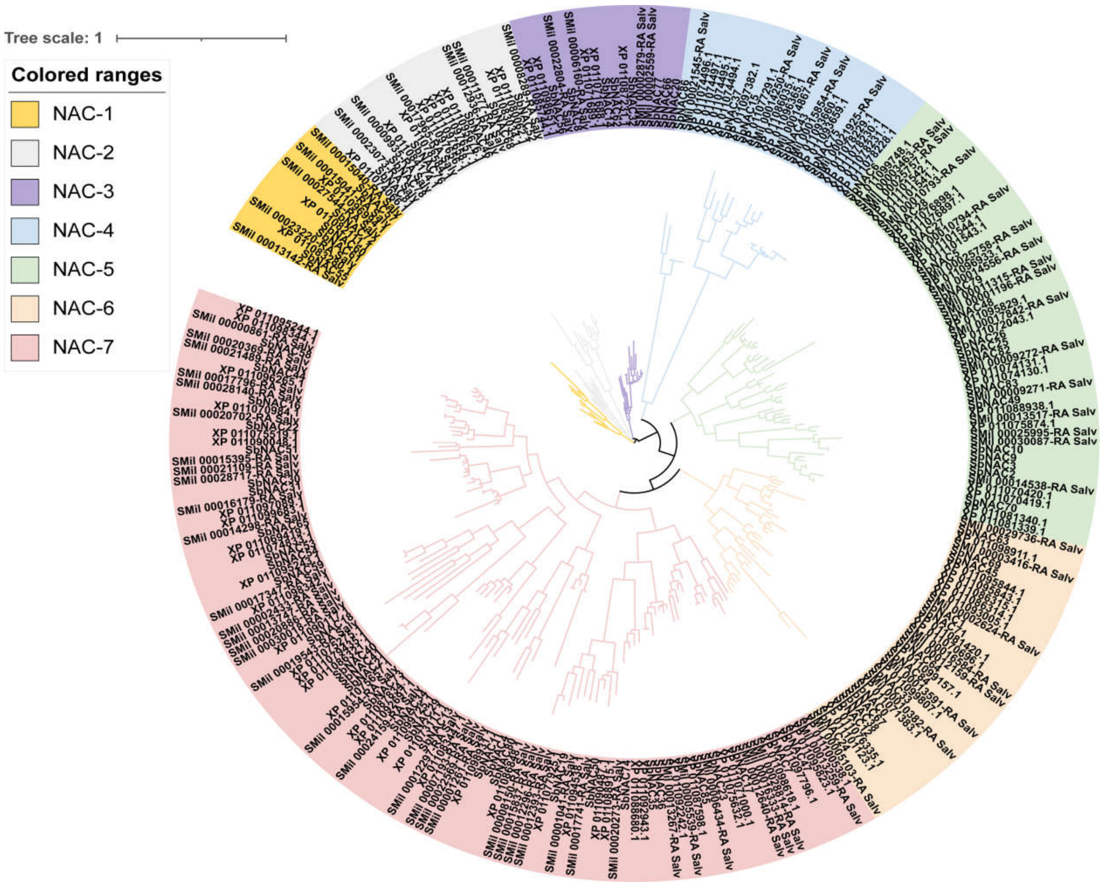

Supplementary Figure S2. Maximum-likelihood phylogenetic tree of *NAC* proteins from *Scutellaria baicalensis* and their orthologs from *Salvia miltiorrhiza* (SMil prefix) and *Sesamum indicum* (XP prefix).The tree was inferred from a full-length amino acid sequence alignment. Seven major clades (designated *NAC*-1 to *NAC*-7) are indicated by colored rings ranging from yellow to pink. Scale bar, 1 amino acid substitution per site. This phylogeny resolves the intra-family relationships and cross-species conservation of the *SbNAC* family, and provides a framework for identifying candidate genes involved in secondary metabolism regulation.

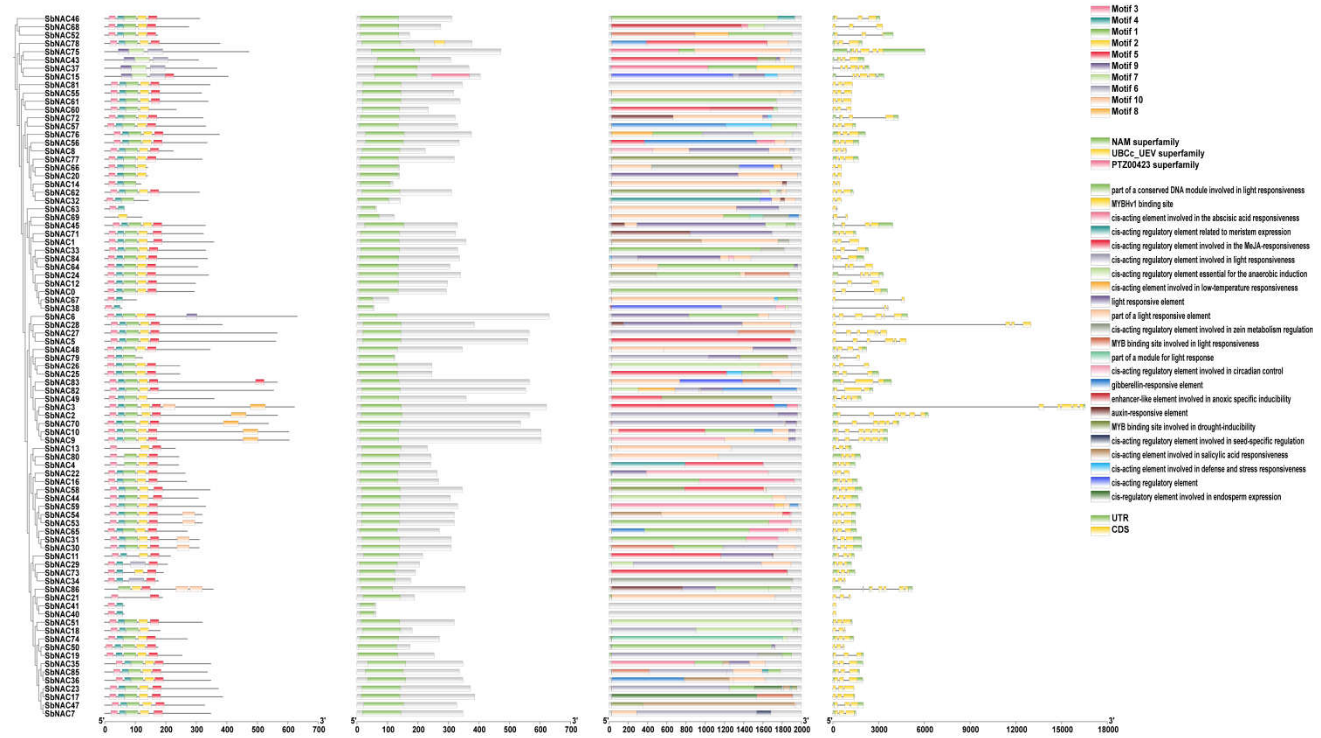

Supplementary Figure S3. Overview of the *SbNAC* gene family. The integrated map illustrates the phylogenetic tree, conserved motifs, protein domain superfamilies, exon-intron structures, and promoter cis-regulatory elements.

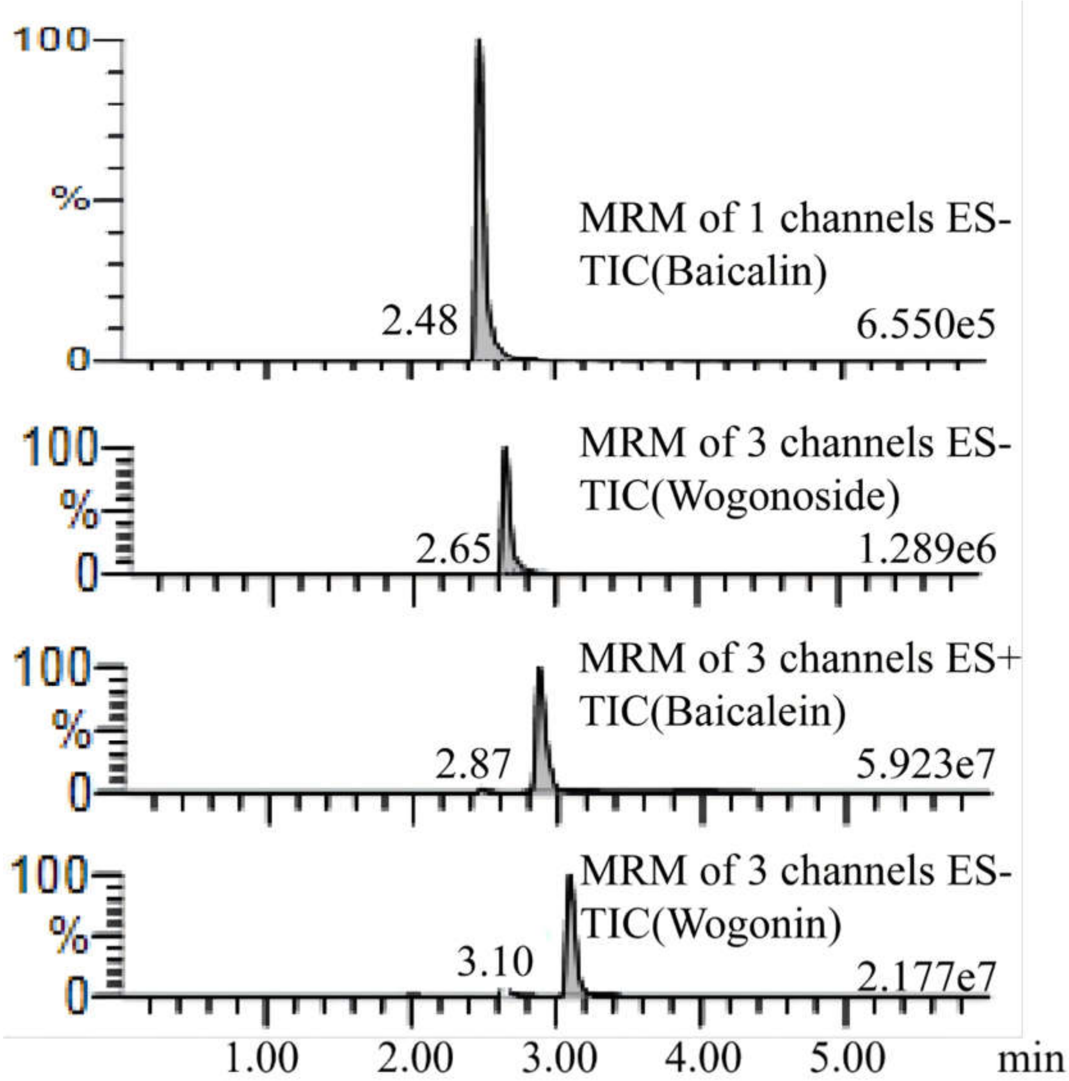

Supplementary Figure S4. TIC chromatogram of four standards

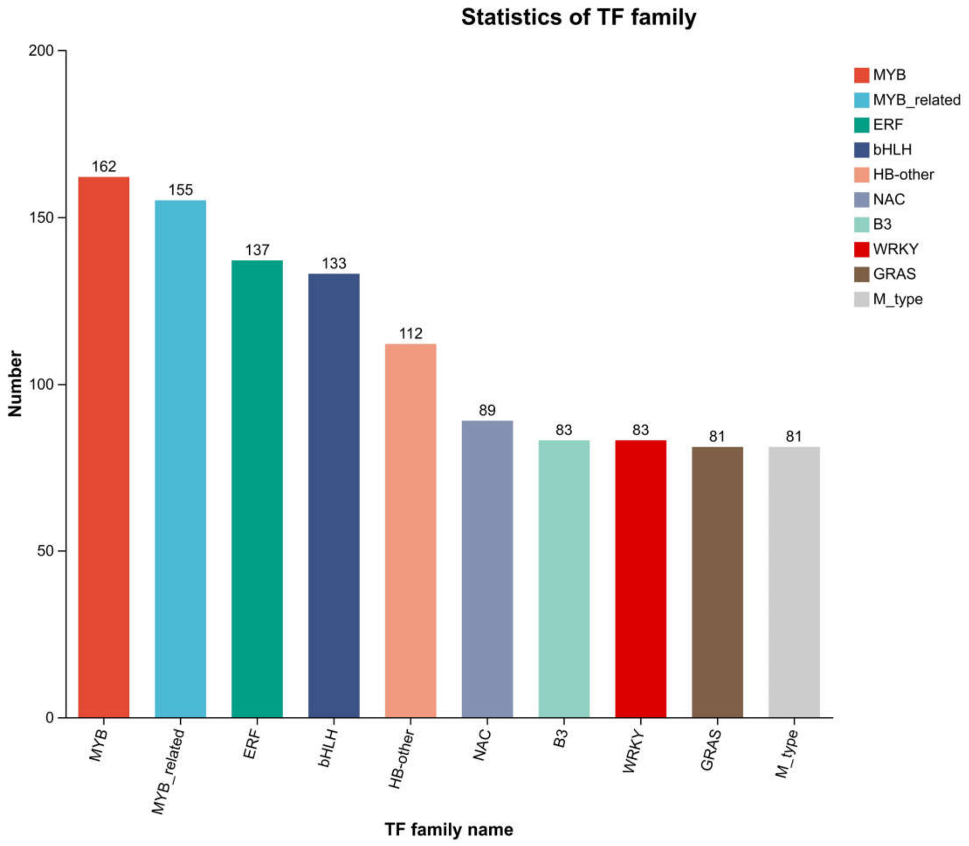

Supplementary Figure S5. Distribution of the top 10 transcription factor (TF) families in the *Scutellaria baicalensis* genome.Bar heights and numerical labels represent the gene count for each family. Colors indicate specific families (see legend). The *NAC* family comprises 89 members, ranking sixth in overall abundance.

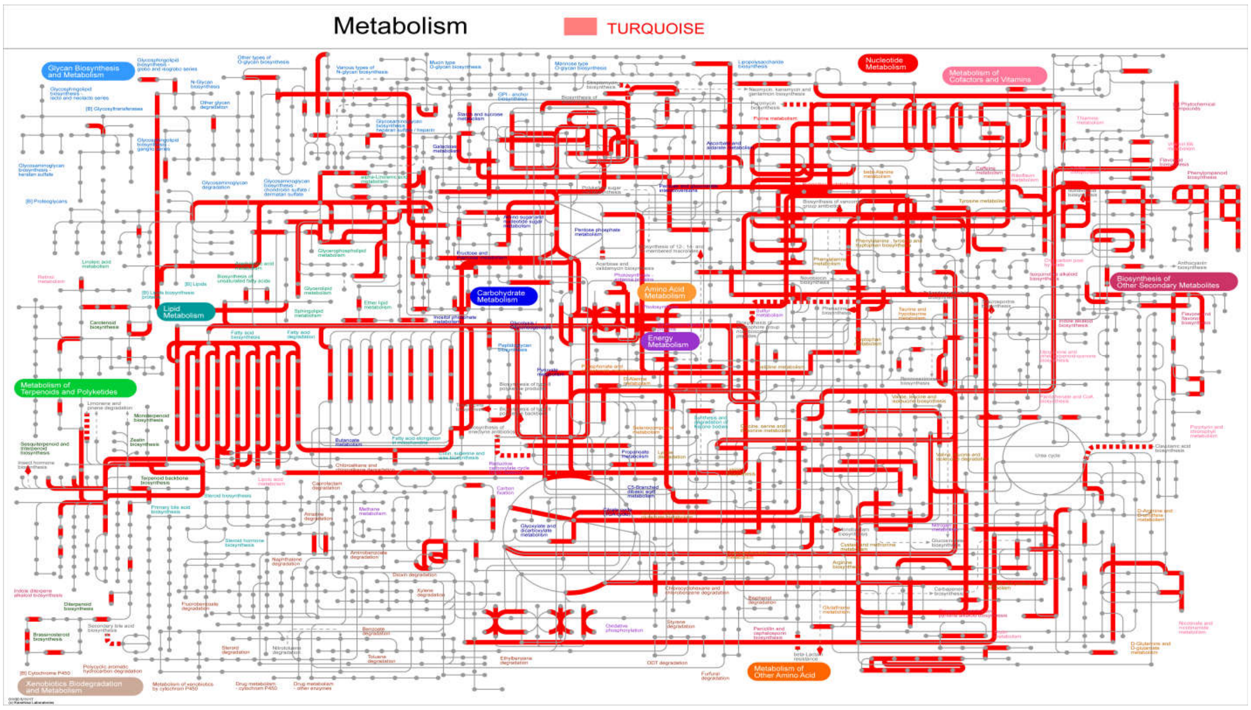

Supplementary Figure S6. Global metabolic pathway mapping of flavonoids biosynthesis in *Scutellaria baicalensis* based on iPath analysis.The circular diagram illustrates the complete metabolic network of *S. baicalensis*. The thick red lines indicate the significantly enriched metabolic pathways associated with the biosynthesis and accumulation of flavonoids, primarily highlighting the central carbon metabolism including glycolysis and the TCA cycle, amino acid metabolism (specifically phenylalanine, tyrosine, and tryptophan biosynthesis), and the downstream phenylpropanoid and flavonoid biosynthesis pathways. Colored rectangular nodes denote major functional categories. The grey lines represent unenriched or background metabolic routes.

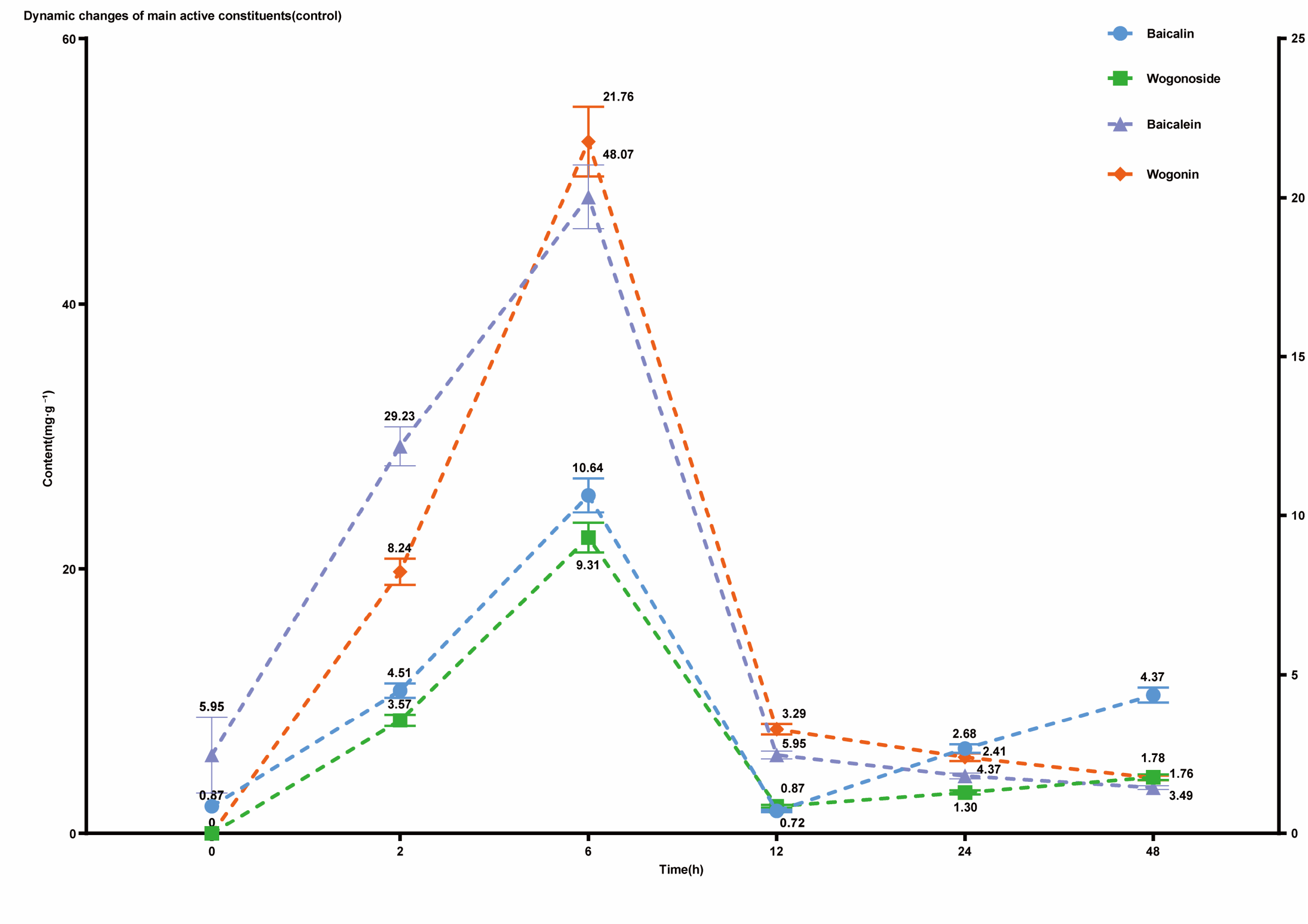

Supplementary Figure S7. Dynamic changes of the main active constituents in the time-matched distilled-water control (CK) of *Scutellaria baicalensis* roots. The control plants were grown and treated in parallel with the 0.5% NaCl-treated plants shown in Figure 2C. Control plants received equal-volume distilled water instead of NaCl, and NaCl was applied only once at the beginning of the salt treatment. Root samples were collected at 0, 2, 6, 12, 24, 48 h after treatment. Baicalin, wogonoside, baicalein, wogonin were quantified by UPLC-MS/MS. The left y-axis shows the contents of baicalin and baicalein, and the right y-axis shows the contents of wogonoside and wogonin. The four curves represent the time-course contents of these flavones in the distilled-water control group. Values are means ± SD (n = 3 pots per treatment, six plants per pot; each replicate was pooled from six plants). The 48 h time point was included only for metabolite profiling. These data are provided as the time-matched control for Figure 2C and are presented separately to avoid overcrowding the main figure and to allow direct comparison with the salt-treatment dynamics in Figure 2C.
